# Supplementary material for: Efficient retrosynthetic planning with MCTS exploration enhanced A* search
Source: Commun Chem. 2024 Mar 7;7:52. doi: 10.1038/s42004-024-01133-2 (PMC10920677; doi:10.1038/s42004-024-01133-2)
Supplement: Supplementary file 2 — Supplementary Information [file 42004_2024_1133_MOESM2_ESM.pdf]

Supplementary Note 1 Optimality of MEEA*

It is known that the tree-search version of A* is guaranteed to find the optimal solution under certain conditions. For the practical computation of Equation 1, $g(s)$ is accumulated through the real interactions, while $h(s)$ is calculated by a heuristic function $\hat{h}(s)$ and each state is evaluated by $\hat{f}(s) = g(s) + \hat{h}(s)$. The optimality of A* holds if $\hat{h}$ is admissible, i.e., $\hat{h}$ never overestimates the cost to the goal. In other words, $\hat{h}(s) \leq h(s)$ holds for all states. However, A* search is shown to be not always optimal for some problems under admissible assumption. A stronger condition called the consistency assumption is required [1, 2].

Assumption 1. *The heuristic function $\hat{h}(s)$ is **consistent** if, for every node s and its successor s', the following inequality holds:*

$$\hat{h}(s) \leq c(s, s', a) + \hat{h}(s'), \tag{S1}$$

where $c(s, s', a)$ is the actual cost of transitioning from s to s' using action a.

Clearly, consistency implies admissibility. Based on the consistency assumption stated in Assumption 1, we can derive Corollary 2 by adding $g(s)$ to both sides of Equation S1.

Corollary 2. *If $\hat{h}$ is consistent, then the $\hat{f}$ value is non-decreasing along any path, which is $\hat{f}(s') \geq \hat{f}(s)$ holds for every node s and its successor s'.*

Notice that if $\hat{h}$ satisfies the consistency assumption, it is also admissible. Therefore, in general, A* search is optimal if the heuristic function $\hat{h}$ is consistent. Due to the exploration nature in Equation 3, if a node is closed by A*, then the node will be closed by MEEA*. Formally, with the help of Corollary 2, we have the following lemma,

Lemma 3. *All nodes closed by A* search are also closed by MEEA*, i.e., $\forall a, \exists b \geq a, C_a^1 \subseteq C_b^2$, where C_a^1 denotes the closed set of A* after a times expansion, and C_b^2 denotes the closed set of MEEA* after b times expansion.*

Next, we proceed to prove Lemma 3. In the first step, both A* search and MEEA* expand the root state s_0 and add its children to the open set. Let O_a^1 denote the open set of A* after a times expansion, and O_b^2 denotes the open set of MEEA* after b times expansion. That is, $C_1^1 \subseteq C_1^2$ and $O_1^1 = O_1^2$. Next, we will prove Lemma 3 using mathematical induction. Assume after a times expansion of A*:

$$C_a^1 = \{s_1^1, s_2^1, \cdots, s_a^1\}, \qquad O_a^1 = \{o_1^1, o_2^1, \cdots, o_p^1\}, \tag{S2}$$

where C_a and O_a are the closed set and opening set correspondingly. MEEA* after b times expansion satisfies that $C_a^1 \subseteq C_b^2$, and

$$C_b^2 = \{s_1^2, s_2^2, \cdots, s_b^2\}, \qquad O_b^2 = \{o_1^2, o_2^2, \cdots, o_q^2\} \tag{S3}$$

Because each open node is a child of a closed node and $C_a^1 \subseteq C_b^2$, we have that

$$\forall s \in O_a^1,\ s \in O_b^2 \ or\ s \in C_b^2 \quad \forall s \in O_b^2,\ s \in O_a^1 \ or\ Anc(s) \in O_a^1, \tag{S4}$$

where $Anc(s)$ is a ancestor state for s. Assume A* expands s^1_{a+1} in the next step, which is

$$s^1_{a+1} = \arg\min\{\hat{f}(s)|s \in O^1_a\}. \tag{S5}$$

If $s^1_{a+1} \in C^2_b$, Lemma 5.3 is established after $a+1$ expansions. If $s^1_{a+1} \in O^2_b$, we first prove that the optimality of s^1_{a+1} is not changed in O^2_b. For state $s \in O^2_b$, there are two cases that need to be discussed:

- $s \in O^1_a$: $\hat{f}(s^1_{a+1}) \le \hat{f}(s)$ according to Equation S5.
- $Anc(s) \in O^1_a$: Based on Corollary 5.2, $\hat{f}(Anc(s)) \le \hat{f}(s)$. Because $Anc(s) \in O^1_a$, $\hat{f}(s^1_{a+1}) \le \hat{f}(Anc(s))$ according to Equation S5. Thus, $\hat{f}(s^1_{a+1}) \le \hat{f}(Anc(s)) \le \hat{f}(s)$.

Therefore, s^1_{a+1} is also the optimal node in O^2_b:

$$s^1_{a+1} = \arg\min\{\hat{f}(s)|s \in O^2_b\}. \tag{S6}$$

While selecting the expanded node for MEEA*, a set of candidate nodes S^{Ca}_{b+1} is sampled from O^2_b using pUCT criterion and $S^{Ca}_{b+1} \in O^2_b$. There are two cases to consider:

- $s^1_{a+1} \in S^{Ca}_{b+1}$: Based on Equation S6, $s^1_{a+1} = \arg\min\{\hat{f}(s)|s \in S^{Ca}_{b+1}\}$ is established because $S^{Ca}_{b+1} \in O^2_b$, and s^1_{a+1} is expanded by MEEA*.
- $s^1_{a+1} \notin S^{Ca}_{b+1}$: Another node $s^2_{b+1} \ne s^1_{a+1}$ is expanded. The children of s^2_{b+1} are added to the opening set, which is $O^2_{b+1} = (O^2_b \setminus \{s^2_{b+1}\}) \cup Child$. According to Corollary 5.2, the $\hat{f}$-values of these newly added children to O^2_{b+1} are greater than or equal to $\hat{f}(s^2_{b+1})$. Therefore, Equation S6 still holds for $b+1$.

$$s^1_{a+1} = \arg\min\{\hat{f}(s)|s \in O^2_{b+1}\}. \tag{S7}$$

Due to the exploratory nature of MCTS, the node s^1_{a+1} will be sampled as a candidate node after a sufficient number of iterations. Assume that $s^1_{a+1} \in S^{Ca}_{b+l}$. In this case, Equation S6 is still satisfied for $b+l$, and s^1_{a+1} is selected to be expanded. Therefore, Lemma 3 is established after $a+1$ expansions. Thus, Lemma 3 holds for any closed node of A* search. The additional expanded nodes in $C^2_{b+l} \setminus C^1_{a+1}$ are the outcomes of the exploration of MEEA*, which may contain the goal state in advance. By leveraging the optimality of A* under Assumption 1, together with Lemma 3, we can prove the optimality of MEEA*, as given by the following theorem.

Theorem 4. *MEEA* is guaranteed to find the optimal solution if $\hat{h}$ is consistent.*

It needs to be noted that consistency assumption is about the heuristic estimation provided by the neural network, and the stored statistics $Q(s,a)$ in MCTS simulations is not necessary to be consistent.

Supplementary Note 2 Introduction to benchmark algorithms

Previous algorithms primarily focused on training more reliable heuristic functions, while search algorithms typically employed MCTS or A*-like search. As illustrated in

Table 1, Retro*, Retro*+, RetroGraph, and BioNavi-NP employ the same A* search algorithm. The cost estimator of Retro*+ and BioNavi-NP are the same as Retro*. The single-step expansion policy of EG-MCTS is borrowed from Retro*.

Supplementary Table 1: Introduction to benchmark algorithms.

Algorithm	Heuristic function	Search algorithm
Retro* [3]	Single-step expansion policy and cost estimator trained using supervised learning.	A* search on an AND-OR tree
Retro*+ [4]	Update the single-step policy using reinforcement learning while keeping the cost estimator the same as Retro*.	Same as Retro*
RetroGraph [5]	Single-step expansion policy and cost estimator trained using reinforcement learning.	Same as Retro*
MCTS [6]	Single-step expansion policy is trained using supervised learning. The fast rollout is used to estimate the cost.	MCTS
EG-MCTS [7]	Single-step expansion policy is borrowed from Retro*+, cost estimator is trained using reinforcement learning.	MCTS
GRASP [8]	Single-step expansion policy and cost estimator trained using reinforcement learning.	MCTS
BioNavi-NP [9]	Single-step expansion policy is trained using supervised learning, cost estimator is borrowed from Retro*.	Same as Retro*

Supplementary Note 3 Network architecture

As shown in Figure 3, there are two neural networks employed in this paper: a policy network in the single-step retrosynthetic model $\mathcal{B}$ and a value network for state evaluation. The architecture of the policy network is identical to Retro* [3] and Retro*+ [4]. The input molecule is represented by its Morgan fingerprint, which is a vector of 2048 dimension. The output is a probability distribution over all available 381302 chemical templates. The policy network is composed of:

- A fully connected layer $[2048, 512]$.
- A batch normalization layer.
- A dropout layer with a dropout rate of 0.3.
- A fully connected layer $[512, 381302]$.

When estimating synthetic cost independently in Equation 5, the architecture of the value network is also identical to Retro* [3] and Retro*+ [4], which is composed of:

- A fully connected layer $[2048, 128]$.
- A ReLU activation layer.
- A dropout layer with a dropout rate of 0.1.
- A fully connected layer $[128, 1]$.

When estimating synthetic cost jointly in Equation 6, the input of the value network consists of multiple molecules. A summation layer is utilized to perform element-wise summation of the extracted features of all molecules, producing a global feature representation. The architecture is composed of:

- A fully connected layer $[2048, 128]$.
- A ReLU activation layer.
- A dropout layer with a dropout rate of 0.1.
- A summation layer to produce global representation $N \times 128 \rightarrow 1 \times 128$.
- A fully connected layer $[128, 1]$.

Supplementary Note 4 Introduction to test datasets

More detailed information about the test datasets is listed in Table 2, including the data size and their practical implications.

Supplementary Table 2: Introduction to the test dataset.

Dataset	Data Size[1]	Data Size [2]	Proportion	Description
USPTO [3]	190	190	100.00%	United States patents
logS [10]	4801	407	8.47%	solubility prediction
BBBP [11]	2039	610	29.92%	blood-brain barrier penetration
ClinTox [12]	1478	628	42.49%	drug compounds
logP [13]	8871	1073	12.10%	hydrophilicity prediction
DPP4 [14]	3933	2322	59.04%	inhibitors of type 2 diabetes
BACE [15]	1513	1163	76.86%	inhibitors of Alzheimer's
Ames [16]	6512	1129	17.34%	Ames mutagenicity
Toxicity LD50 [17]	7413	872	11.76%	Toxicity prediction
SVS [18]	5450	2916	53.50%	sequence-based virtual screening [3]

[1]Number of molecules in the original dataset.

[2]Number of molecules after filtering.

[3]Investigation on the interaction between proteins and ligand molecules, and inhibition of protein-protein interactions.

Supplementary Note 5 Success rate on USPTO benchmark with different single-step model calls.

As shown in Table 3, although MEEA* exhibits inferior performance compared to GRASP and RetroGraph initially, its performance improves progressively with an increase in search iterations, surpassing GRASP and RetroGraph eventually. GRASP is built on MCTS, and its exploratory nature compels it to allocate iterations to explore non-optimal branches. Conversely, RetroGraph, an A*-like algorithm, is trapped in local optimal branches due to the lack of enough exploration. MEEA* is a combination of MCTS and A*, overcoming the aforementioned shortcomings of both, thereby achieving the best performance.

Supplementary Table 3: The success rate on USPTO benchmark with different single-step model calls.

Iterations	100	200	300	400	500
Retro*+ [4]	71.05%	85.26%	88.95%	90.00%	91.05%
GRASP [8]	90.52%	97.89%	97.89%	98.42%	98.94%
RetroGraph [5]	88.42%	97.89%	98.95%	99.47%	99.47%
MEEA*	88.95%	95.79%	98.42%	99.47%	100.0%

Supplementary Note 6 Ablation study on path consistency

With the single-step expansion policy of Retro*+ as the initial model, the policy is updated with the newly generated synthetic pathways. The cost estimator is replaced with a neural network that can predict the synthesis costs of multiple molecules simultaneously. The training process may or may not include PC constraints. As reported in Table 4, with the guidance of the updated policy and the cost estimator trained under the PC constraint, the overall success rate of MEEA* on the ten datasets has significantly improved from 65.14% to 76.27%. When updating the policy alone without incorporating the PC constraint in cost estimator training, a notable 2.59% increase in success rate is observed. Correspondingly, when the policy remains unaltered, but the cost estimator is trained with the PC constraint, it results in a substantial 8.50% increment in the success rate. The results of the ablation experiments indicate that both updated policy and regularized cost estimator contribute to performance improvement, but PC contributes more significantly.

Supplementary Table 4: Ablation study on path consistency.

		Without PC		With PC	
Dataset	MEEA*[1]	Initial policy[2]	Updated policy[3]	Initial policy	Updated policy
USPTO [3]	**100.0%**	98.84%	96.84%	92.63%	94.74%
logS [10]	73.22%	74.44%	75.18%	78.13%	**80.34%**
BBBP [11]	57.70%	56.69%	60.32%	62.46%	**66.88%**
ClinTox [12]	50.00%	50.80%	51.27%	57.17%	**60.51%**
logP [13]	65.24%	65.24%	67.94%	72.23%	**73.72%**
DPP4 [14]	83.63%	83.94%	89.75%	91.26%	**96.04%**
BACE [15]	40.15%	40.50%	40.93%	61.99%	**56.32%**
Ames [16]	68.29%	68.82%	71.30%	75.64%	**78.74%**
Toxicity LD50 [17]	66.28%	66.05%	63.65%	75.23%	**72.94%**
SVS [18]	60.19%	60.39%	63.99%	68.76%	**73.01%**
Total	65.14%	65.45%	68.04%	73.95%	**76.27%**

[1]Policy and cost estimation models of MEEA* are borrowed from Retro*+.

[2]Initial policy is the policy model provided by Retro*+.

[3]Updated policy is the policy model updated from the initial policy.

Supplementary Note 7 Exploring different single-step expansion model on the effectiveness of MEEA*

MEEA* can be combined with different single-step expansion models. According to Table 5, when the single-step expansion model of Retro* is combined with MEEA*, the success rate is improved from 54.18% to 58.92%. When the single-step expansion model of Retro*+ is combine with MEEA*, the success rate is improved from 60.50% to 62.20%. What's more, employing a better single-step expansion model can also enhance the performance of MEEA*. The policy model of Retro*+ is refined from that of Retro*, and our policy model is refined from the policy model of Retro*+. With each iteration of refinement, the results of the MEEA* search assisted by the single-step expansion model are demonstrated to improve successively. The success rate of MEEA* assist by the single-step model provided by Retro*, Retro*+, and ours, is 58.92%, 62.20%, and 67.93%, respectively.

Supplementary Table 5: Success rate for MEEA* with different expansion policy. (The cost estimator for Retro*, Retro*+, and MEEA* is identical.)

Dataset	Retro* [3]	MEEA* [1]	Retro*+ [4]	MEEA* [2]	MEEA* [3]
USPTO [3]	86.84%	96.32%	91.05%	100.0%	96.84%
logS [10]	67.08%	73.96%	69.29%	73.22%	74.20%
BBBP [11]	47.87%	55.25%	52.46%	57.70%	59.84%
ClinTox [12]	38.69%	45.06%	43.15%	50.00%	51.75%
logP [13]	53.96%	60.58%	61.14%	65.24%	67.19%
DPP4 [14]	68.52%	72.31%	78.59%	83.63%	89.79%
BACE [15]	33.71%	35.94%	38.35%	40.15%	41.27%
Ames [16]	57.40%	62.53%	63.51%	66.61%	70.68%
Toxicity LD50 [17]	55.39%	61.12%	59.98%	66.28%	64.22%
SVS [18]	50.14%	53.98%	55.93%	60.19%	63.89%
Total	54.18%	58.92%	60.50%	62.20%	67.93%

[1]Single-step expansion policy is borrowed from Retro*.

[2]Single-step expansion policy is borrowed from Retro*+.

[3]Single-step expansion policy is trained by ours.

Supplementary Note 8 Investigation on MCTS simulation times K_{MCTS}

MCTS simulation times K_{MCTS} is a set to balance the influence of A* search and MCTS on MEEA*. When K_{MCTS} is relatively small, MEEA* approaches to MCTS. When K_{MCTS} is quite large, MEEA* closely resembles A* search. As shown in Figure 1, both extreme scenarios exhibit a reduction in performance. Appropriate K_{MCTS} achieves a better balance between exploration and exploitation, resulting in

improved performance. The results indicate that there is a wide range for K_{MCTS} to make MEEA* perform well.

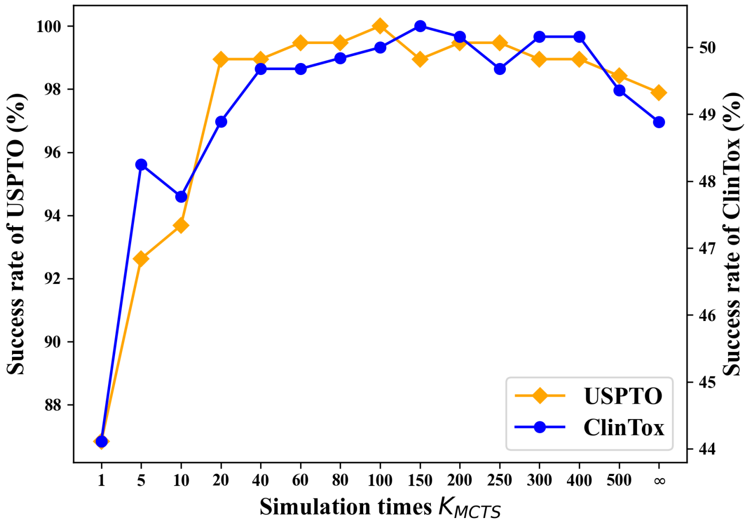

Supplementary Figure 1: Success rate of MEEA* with different K_{MCTS}.

Supplementary Note 9 Success rate with different search time

Detailed comparisons of success rates under different limits on the search time are presented in Figure 2 and Table 6 below. For molecules that can be solved easily by the well-trained heuristics, A* search is the most efficient algorithm, because A* has less additional unnecessary exploration. Hence, under a small limit on the search time, A* achieves the highest success rate in Figure 2 and Table 6, while MEEA* is comparable to or slightly worse than A*. For the remaining challenging molecules, all algorithms need more time to find a feasible solution. MEEA* efficiently identified feasible solutions for all molecules in the shortest time by incorporating appropriate exploration. A* is trapped in non-optimal branches and it requires more time than MEEA* to escape. The time required by MCTS is the longest because of its compulsory too much exploration. All algorithms are guided by the same heuristics and are capable of finding the feasible solution. Without caching the calls to the single-step model, more time is required by Retro*+ [19].

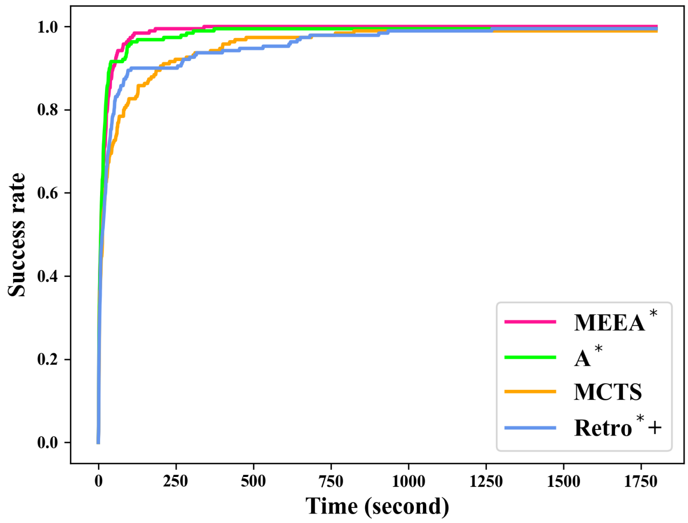

Supplementary Figure 2: Comparison of success rates with different search time.

Supplementary Table 6: Success rates with different search time.

Time(s)	10	30	60	120	240	360	480	600	1200	1800
Retro*+	47.37%	68.95%	83.16%	90.00%	90.00%	93.68%	94.74%	96.84%	98.95%	99.47%
MCTS	44.74%	65.79%	74.21%	82.63%	91.58%	93.68%	97.37%	97.37%	98.95%	98.95%
A*	**56.32%**	**85.79%**	91.58%	96.32%	97.37%	98.95%	99.47%	99.47%	99.47%	99.47%
MEEA*	**56.32%**	81.05%	**93.16%**	**98.42%**	**99.47%**	**100.0%**	**100.0%**	**100.0%**	**100.0%**	**100.0%**

Supplementary Note 10 Distribution of USPTO benchmark

The distribution of the USPTO dataset is visualized in Figure 3, where the t-SNE algorithm is employed to reduce the dimensionality of the MorganFingerprint.

Supplementary Note 11 An example of the synthesis of a natural product

Figure 4 (a) & (b) present a synthetic pathway for a real NP provided by BioNavi-NP and MEEA*-PC. The presence of exploratory features in our algorithm has enabled the discovery of a shorter synthetic pathway.

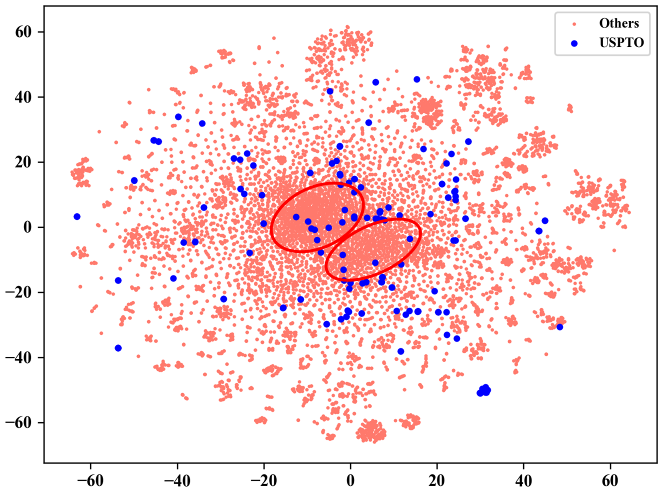

Supplementary Figure 3: Distribution of molecules in USPTO benchmark and other datasets. Molecule's Morgan Fingerprint is reduced to two dimensions using the t-SNE algorithm.

Supplementary Note 12 Examples of the synthesis of drug molecules

Paxlovid is an oral antiviral medication authorized by the the United States Food and Drug Administration (FDA) in 2021 for the treatment of COVID-19. The CAS number of Paxlovid is 2628280-40-8. Fostemsavir is an HIV attachment inhibitor and was approved in 2020 by the FDA. Enarodustat was approved by the Japan Pharmaceuticals and Medical Devices Agency (PMDA) in 2020 for the treatment of anemia associated with chronic kidney disease. Pacritinib is an anti-cancer medication used to treat myelofibrosis authorized by the FDA in 2022. Oteseconazole is approved by the FDA in 2022 for the treatment of recurrent vulvovaginal candidiasis. MEEA* has successfully identified the synthesis pathway for the above five drugs, and the synthetic plans are illustrated in Figure 5.

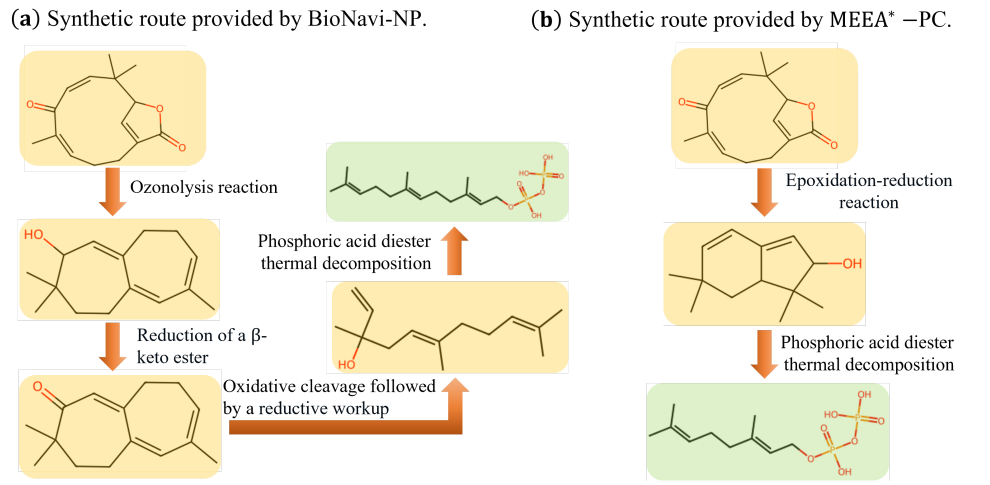

Supplementary Figure 4: Synthetic route provided by BioNavi-NP and MEEA*-PC for natural product C/C1=C\\CCC2=CC(OC2=O)C(C)(C)/C=C/C1=O. MEEA*-PC has found a shorter synthetic pathway.

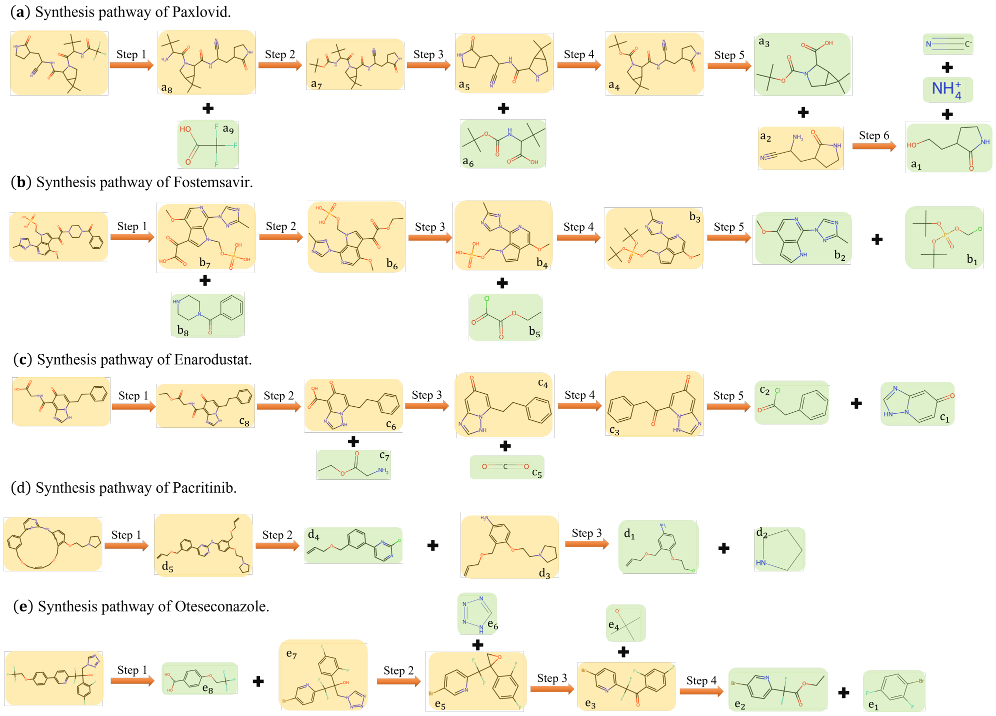

Supplementary Figure 5: Synthetic route for drug molecules provided by MEEA*: (a) Paxlovid (2628280-40-8); (b) Fostemsavir (864953-29-7); (c) Enarodustat (1262132-81-9); (d) Pacritinib (937272-79-2); (e) Oteseconazole (1340593-59-0).

Taking Paxlovid as an example, building block m_1 undergoes a chemical reaction with the ionic compound $C \equiv N^-$ and $NH4^+$ to yield product m_2, which is an amide derivative. The carboxylic acid group of m_3 is replaced by the cyanide group of m_2, resulting in the formation of a new amide functional group. Then, m_4 undergoes a structural rearrangement to form the molecule m_5, which undergoes a cyclization reaction with m_6 to yield m_7. Dehydration reaction of m_7 produces m_8. Finally, m_8 and m_9 undergo a dehydration condensation reaction to form the Paxlovid successfully.

Supplementary References

[1] Dechter, R., Pearl, J.: Generalized best-first search strategies and the optimality of a*. Journal of the ACM (JACM) **32**(3), 505–536 (1985)

[2] Russell, S.J.: Artificial Intelligence a Modern Approach. Pearson Education, Inc., Upper Saddle River, N.J. :Prentice Hall (2010)

[3] Chen, B., Li, C., Dai, H., Song, L.: Retro*: learning retrosynthetic planning with neural guided a* search. In: International Conference on Machine Learning, pp. 1608–1616 (2020). PMLR

[4] Kim, J., Ahn, S., Lee, H., Shin, J.: Self-improved retrosynthetic planning. In: International Conference on Machine Learning, pp. 5486–5495 (2021). PMLR

[5] Xie, S., Yan, R., Han, P., Xia, Y., Wu, L., Guo, C., Yang, B., Qin, T.: Retrograph: Retrosynthetic planning with graph search. In: Proceedings of the 28th ACM SIGKDD Conference on Knowledge Discovery and Data Mining, pp. 2120–2129 (2022)

[6] Segler, M.H., Preuss, M., Waller, M.P.: Planning chemical syntheses with deep neural networks and symbolic ai. Nature **555**(7698), 604–610 (2018)

[7] Hong, S., Zhuo, H.H., Jin, K., Shao, G., Zhou, Z.: Retrosynthetic planning with experience-guided monte carlo tree search. Communications Chemistry **6**(1), 120 (2023)

[8] Yu, Y., Wei, Y., Kuang, K., Huang, Z., Yao, H., Wu, F.: Grasp: Navigating retrosynthetic planning with goal-driven policy. In: Advances in Neural Information Processing Systems (2022)

[9] Zheng, S., Zeng, T., Li, C., Chen, B., Coley, C.W., Yang, Y., Wu, R.: Deep learning driven biosynthetic pathways navigation for natural products with bionavi-np. Nature Communications **13**(1), 3342 (2022)

[10] Xiong, G., Wu, Z., Yi, J., Fu, L., Yang, Z., Hsieh, C., Yin, M., Zeng, X., Wu, C., Lu, A., *et al.*: Admetlab 2.0: an integrated online platform for accurate and comprehensive predictions of admet properties. Nucleic Acids Research **49**(W1), 5–14 (2021)

[11] Martins, I.F., Teixeira, A.L., Pinheiro, L., Falcao, A.O.: A bayesian approach to in silico blood-brain barrier penetration modeling. Journal of chemical information and modeling **52**(6), 1686–1697 (2012)

[12] Gayvert, K.M., Madhukar, N.S., Elemento, O.: A data-driven approach to predicting successes and failures of clinical trials. Cell chemical biology **23**(10), 1294–1301 (2016)

[13] Cheng, T., Zhao, Y., Li, X., Lin, F., Xu, Y., Zhang, X., Li, Y., Wang, R., Lai, L.: Computation of octanol- water partition coefficients by guiding an additive model with knowledge. Journal of chemical information and modeling **47**(6), 2140–2148 (2007)

[14] Hermansyah, O., Bustamam, A., Yanuar, A.: Virtual screening of dpp-4 inhibitors using qsar-based artificial intelligence and molecular docking of hit compounds to dpp-8 and dpp-9 enzymes (2020)

[15] Subramanian, G., Ramsundar, B., Pande, V., Denny, R.A.: Computational modeling of β-secretase 1 (bace-1) inhibitors using ligand based approaches. Journal of chemical information and modeling **56**(10), 1936–1949 (2016)

[16] Hansen, K., Mika, S., Schroeter, T., Sutter, A., Ter Laak, A., Steger-Hartmann, T., Heinrich, N., Muller, K.-R.: Benchmark data set for in silico prediction of ames mutagenicity. Journal of chemical information and modeling **49**(9), 2077–2081 (2009)

[17] Wu, K., Wei, G.-W.: Quantitative toxicity prediction using topology based multitask deep neural networks. Journal of chemical information and modeling **58**(2), 520–531 (2018)

[18] Shen, L., Feng, H., Qiu, Y., Wei, G.-W.: Svsbi: Sequence-based virtual screening of biomolecular interactions. arXiv preprint arXiv:2212.13617 (2022)

[19] Maziarz, K., Tripp, A., Liu, G., Stanley, M., Xie, S., Gaiński, P., Seidl, P., Segler, M.: Re-evaluating retrosynthesis algorithms with syntheseus. arXiv preprint arXiv:2310.19796 (2023)
