# Supplementary material for: Efficient retrosynthetic planning with MCTS exploration enhanced A* search
Source: Commun Chem. 2024 Mar 7;7:52. doi: 10.1038/s42004-024-01133-2 (PMC10920677; doi:10.1038/s42004-024-01133-2)
Supplement: Supplementary file 3 — Description of Additional Supplementary Files [file 42004_2024_1133_MOESM3_ESM.pdf]

Description of Additional Supplementary Files

File name: Supplementary Data 1

Description: Numerical source data for Figure2 (c)

File name: Supplementary Data 2

Description: Numerical source data for Figure2 (d)

File name: Supplementary Data 3

Description: Numerical source data for Figure4 (a)

File name: Supplementary Data 4

Description: Numerical source data for Figure4 (b)

File name: Supplementary Data 5

Description: Numerical source data for Figure4 (c)
